# Expression, Purification, and Biophysical Characterization of a Secreted Anthrax Decoy Fusion Protein in *Nicotiana benthamiana*

**DOI:** 10.3390/ijms18010089

**Published:** 2017-01-04

**Authors:** Kalimuthu Karuppanan, Sifti Duhra-Gill, Muchena J. Kailemia, My L. Phu, Carlito B. Lebrilla, Abhaya M. Dandekar, Raymond L. Rodriguez, Somen Nandi, Karen A. McDonald

**Affiliations:** 1 Department of Chemical Engineering, University of California, Davis, CA 95616, USA; kkaruppanan@ucdavis.edu (K.K.); sdgill@ucdavis.edu (S.D.-G.); 2 Department of Chemistry, University of California, Davis, CA 95616, USA; jkmuchena@ucdavis.edu (M.J.K.); cblebrilla@ucdavis.edu (C.B.L.); 3 Department of Plant Sciences, University of California, Davis, CA 95616, USA; mlphu@ucdavis.edu (M.L.P.); amdandekar@ucdavis.edu (A.M.D.); 4 Department of Molecular & Cellular Biology, University of California, Davis, CA 95616, USA; rlrodriguez@ucdavis.edu (R.L.R.); snandi@ucdavis.edu (S.N.); 5 Department of Biochemistry and Molecular Medicine, University of California, Davis, CA 95616, USA

**Keywords:** transient protein expression, *Nicotiana benthamiana*, apoplast wash fluid, anthrax decoy fusion protein, *N*-glycosylation

## Abstract

Anthrax toxin receptor-mediated drug development for blocking anthrax toxin action has received much attention in recent decades. In this study, we produced a secreted anthrax decoy fusion protein comprised of a portion of the human capillary morphogenesis gene-2 (*CMG2*) protein fused via a linker to the fragment crystallizable (Fc) domain of human immunoglobulin G1 in *Nicotiana benthamiana* plants using a transient expression system. Using the Cauliflower Mosaic Virus (*CaMV*) *35S* promoter and co-expression with the p19 gene silencing suppressor, we were able to achieve a high level of recombinant CMG2-Fc-Apo (rCMG2-Fc-Apo) protein accumulation. Production kinetics were observed up to eight days post-infiltration, and maximum production of 826 mg/kg fresh leaf weight was observed on day six. Protein A affinity chromatography purification of the rCMG2-Fc-Apo protein from whole leaf extract and apoplast wash fluid showed the homodimeric form under non-reducing gel electrophoresis and mass spectrometry analysis confirmed the molecular integrity of the secreted protein. The *N*-glycosylation pattern of purified rCMG2-Fc-Apo protein was analysed; the major portion of *N*-glycans consists of complex type structures in both protein samples. The most abundant (>50%) *N*-glycan structure was GlcNAc_2_(Xy_l_)Man_3_(Fuc)GlcNAc_2_ in rCMG2-Fc-Apo recovered from whole leaf extract and apoplast wash fluid. High mannose *N*-glycan structures were not detected in the apoplast wash fluid preparation, which confirmed the protein secretion. Altogether, these findings demonstrate that high-level production of rCMG2-Fc-Apo can be achieved by transient production in *Nicotiana benthamiana* plants with apoplast targeting.

## 1. Introduction

Anthrax is a lethal infection that occurs when *Bacillus anthracis* endospores enter the body through inhalation or a cut in the skin [1,2]. It is a zoonotic disease which is primarily associated with grazing herbivores and domestic animals [3]. While there are no known cases of anthrax transmission between humans, infections can occur through contact with infected animals or animal products, and the associated condition has been referred to as “wool sorters disease” due to exposure to anthrax spores in the wool of contaminated sheep [4,5]. The infection of anthrax disease is caused by inhalation of dormant endospores, which are resistant to heat, drying, gamma radiation, ultraviolet light, and many disinfectants [6]. Their dormancy and hardiness have allowed anthrax endospores to be developed as biological warfare agents [7,8]. Letters containing anthrax spores killed five people in the United States (US) and infected more than a dozen in 2001 [9]. It is estimated that there are 20,000 to 100,000 new human cases of anthrax disease worldwide each year [10].

Pathogenesis of anthrax infection is initiated through endospore germination from spore to a vegetative organism which occurs inside host macrophages. This progression is initiated when endospore receptors detect both amino acid and purine nucleoside germinants [11]. Carbon dioxide levels in blood and tissue, as well as physiologic body temperature, contribute to this development by triggering the production of main virulence factors [12]. Anthrax toxin consists of three distinct proteins; protective antigen (PA), edema factor (EF), and lethal factor (LF) [13,14]. The first stage of toxin entry into the host cell occurs when PA binds to a receptor on the surface of the target cell. Two closely related host cell receptors have been identified, tumor endothelial marker-8 (TEM8) [15] and capillary morphogenesis gene-2 protein (CMG2) [16]; these receptors bind PA with high affinity [17]. PA is proteolytically-cleaved and one of the cleavage fragments oligomerizes into membrane inserting ring-shaped heptamers that bind the EF and LF components, allowing endocytosis of the toxic complex into mammalian cells [18].

Anthrax toxin receptor-mediated drug development for blocking anthrax intoxication has received considerable attention in recent decades. The CMG2 domain is a key receptor mediating anthrax toxin lethality and it has high binding affinity to PA domain. CMG2 is a type I transmembrane protein which includes a signal peptide that directs it to the endoplasmic reticulum during synthesis, an extracellular von Willebrand factor A domain, an Ig-like domain, a cytoplasmic tail, and a transmembrane helix [19]. Recombinant soluble CMG2 has confirmed potency against anthrax toxin [20]. Also, compared with monoclonal antibodies, the soluble CMG2 domain can bind both wild-type and epitope-mutant forms of PA [21]. However, in vivo studies reveal that soluble CMG2 has a short half-life, which is a drawback for its development as a potential anthrax therapeutic or prophylactic [22]. The recent development of protein engineering shows the fusion protein is a promising technology that can be used to improve serum half-life of recombinant proteins and can be an alternative to the existing technology [23].

Plants provide a viable option to mammalian cell cultures for the production of therapeutic biologics, allowing for linearly scalable, cost-effective, and safe production of recombinant proteins. Tobacco leaves are an efficient bioreactor for protein production since tobacco is a non-feed/food crop with a high biomass yield [24]. In this report, the recombinant human CMG2-Fc-Apo fusion protein was transiently produced in *Nicotiana benthamiana* plants under the control of the Cauliflower Mosaic Virus (*CaMV*) 35S constitutive promoter together with the p19 gene silencing suppressor. The production kinetics of this protein were determined by extracting biomass at different time points post-infiltration. Recovery of rCMG2-Fc-Apo from apoplast wash fluid was also investigated. Purification of rCMG2-Fc-Apo from extracted leaf biomass and apolast wash fluid was achieved by protein-A affinity chromatography, and biophysical properties and site-specific *N*-glycosylation of purified rCMG2-Fc-Apo protein were evaluated.

## 2. Results

### 2.1. Gene Construct and Binary Vector Design for Recombinant CMG2-Fc-Apo (rCMG2-Fc-Apo) Protein

**Figure 1:**
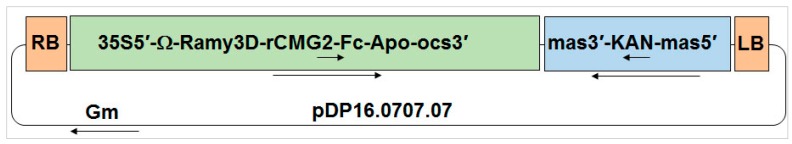
Schematic representation of binary plasmid (pDP16.0707.07) coding for the rCMG2-Fc-Apo protein. RB: right border; LB: left border; 35S: Cauliflower Mosaic Virus (*CaMV*) promoter; *Ramy3D*: coding sequence for the rice α-amylase 3D gene signal peptide; Ω:Ω sequence; *rCMG2-Fc-Apo*: gene coding for the anthrax decoy fusion protein with intact glycosylation site on Fc region; *ocs*: octopine synthase terminator; mas5′ and mas3′: transcription initiation and termination sequences, respectively, from the mannopine synthase gene of *Agrobacterium tumefaciens*; KAN: gene coding for resistance to the antibiotic kanamycin, the arrows indicate transcription direction from 5′ to 3′ ends.

*Nicotiana benthamina* plant codon-optimized fragment of the human CMG2 domain was fused to the Fc domain of human IgG1 using two serines and a hinge region as a fusion protein linker. To secrete the rCMG2-Fc-Apo protein to the *Nicotiana benthamiana* apoplast, the rice α-amylase 3D gene signal peptide (Ramy3D) signal peptide was included on the *N*-terminal region of CMG2 domain. Also, the Ω leader sequence was included to enhance translation and transient protein production. Expression of this protein was accomplished under the control of *CaMV 35S* promoter and octopine synthase (*ocs*) terminator. The clonal selection was achieved by marker-assisted selection using a kanamycin resistance gene on this binary vector (Figure 1).

### 2.2. Production Kinetics of rCMG2-Fc-Apo Protein

**Figure 2:**
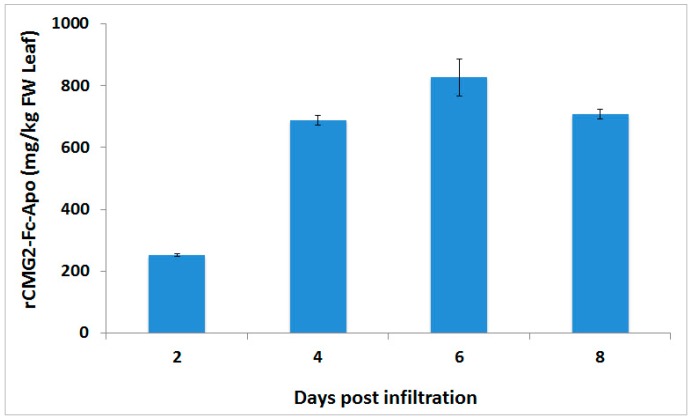
Production kinetics of rCMG2-Fc-Apo protein. Transient production of the rCMG2-Fc-Apo protein on a leaf fresh weight (FW) basis in *Nicotiana benthamiana* plants was measured by ELISA. The p19 gene silencing suppressor was co-expressed to improve the rCMG2-Fc-Apo protein expression. At different time points post-infiltration, entire leaves from an infiltrated plant batch (three plants/batch/data point) were cut at the petioles and harvested and rCMG2-Fc-Apo levels were determined from combined leaf biomass. Error bars were determined from propagation of standard errors calculated from triplicate technical assays performed.

Transient production of the rCMG2-Fc-Apo protein in *Nicotiana benthamiana* was measured by ELISA using Protein-A as a capture molecule and anti-human IgG as a detection antibody (Figure 2). To determine the production kinetics, the expression level of rCMG2-Fc-Apo was estimated at different post-infiltration time points. The production kinetics were determined over eight days post-infiltration on a two-day interval. The results showed that the mass of rCMG2-Fc-Apo per leaf fresh weight peaked at day six post-infiltration at about 800 mg/kg fresh weight (FW), after which time the production started to decline. These results suggested that day six was the optimal time for transient production of rCMG2-Fc-Apo protein in *Nicotiana benthamiana* plants.

### 2.3. Malate Dehydrogenase (MDH) Activity Assay

**Figure 3:**
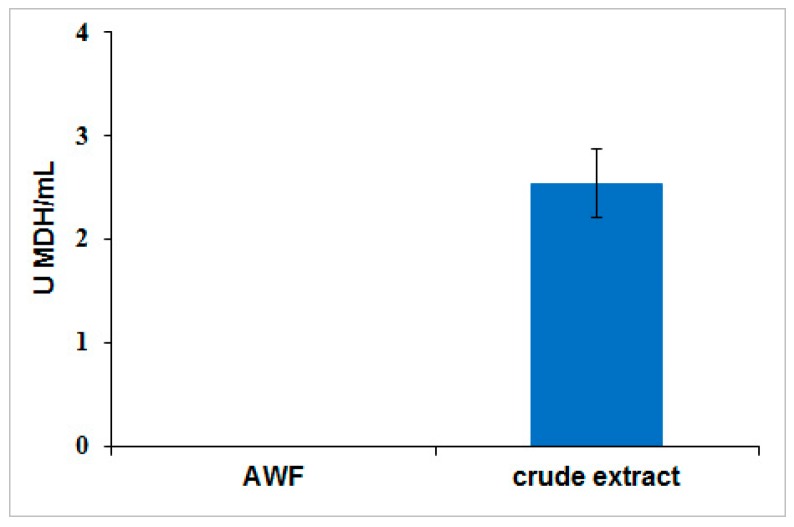
Malate dehydrogenase enzymatic assay. Malate dehydrogenase (MDH) enzyme concentration in whole leaf extract and apoplast wash fluid was monitored by measuring depletion of NADH at 340 nm over 5 min at 25 °C and pH 7.4. The whole leaf extract was diluted for the data normalization to have an equal amount of biomass used for MDH assay samples obtained from the crude extract and the apoplast wash fluid. Error bars were determined from propagation of standard errors calculated from triplicate technical assays performed.

Contamination of intracellular proteins in the apoplast fluid was estimated by measuring an intracellular enzyme marker, malate dehydrogenase (MDH). This enzyme is part of Kreb’s cycle, catalyzing the reversible oxidation of malate to oxaloacetate. Since the reaction resides within the matrix of the mitochondrion, it is an efficient intracellular marker. The apoplast wash fluid from post-agroinfiltrated *Nicotiana benthamiana* leaves was recovered for the MDH enzyme assay. The results showed that no measurable MDH activity was observed in the apoplast wash fluid when compared with the whole leaf extract (Figure 3). This result confirmed that the recovered protein from apoplast fluid is free from intracellular contaminants.

### 2.4. Protein Purification

**Figure 4:**
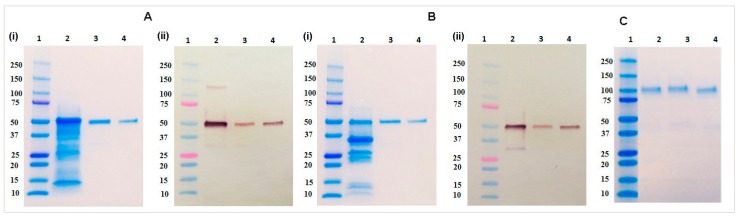
SDS-PAGE, immunoblot, and non-reducing gel analysis. (**A**) Whole leaf extracts and (**B**) apoplast wash fluid. (**i**): SDS-PAGE of whole leaf extract. Lane 1 Precision Plus Protein^TM^ Dual Color Standards (Bio-Rad); Lane 2 crude leaf extract (20 µg); Lane 3 purified rCMG2-Fc-Apo (500 ng); Lane 4 CMG2-Fc standard (500 ng); (**ii**): Immunoblot analysis of whole leaf extract. Lane 1 Precision Plus Protein^TM^ Dual Color Standards (Bio-Rad); Lane 2 crude leaf extract (10 µg); Lane 3 purified rCMG2-Fc-Apo (100 ng); Lane 4 CMG2-Fc standard (100 ng); (**C**) Non-reducing gel. Lane 1 Precision Plus Protein^TM^ Dual Color Standards (Bio-Rad); Lane 2 purified rCMG2-Fc-Apo from whole leaf extract (500 ng); Lane 3 purified rCMG2-Fc-Apo from apoplast wash fluid (500 ng); Lane 4 CMG2-Fc standard (500 ng).

The rCMG2-Fc-Apo protein from whole leaf extract and apoplast wash fluid of *Nicotiana benthamiana* plant were purified using Protein A affinity chromatography. The identity of rCMG2-Fc-Apo protein were validated by SDS-PAGE and immunoblotting analysis. The size of the purified rCMG2-Fc-Apo protein band was shown to be around 50 kDa under reducing conditions in both whole leaf extract and apoplast wash fluid (Figure 4A(i),B(i)). Also, immunoblot analysis by Fc domain detection on the rCMG2-Fc-Apo protein shows the molecule identity (Figure 4A(ii),B(ii)). Similarly, the size of purified rCMG2-Fc-Apo protein from whole leaf extract and apoplast wash fluid was shown to be around 100 kDa under non-reducing conditions (Figure 4C); this result confirms the integrity of Fc domain homodimerization.

### 2.5. Mass Spectrometry Analysis

**Figure 5:**
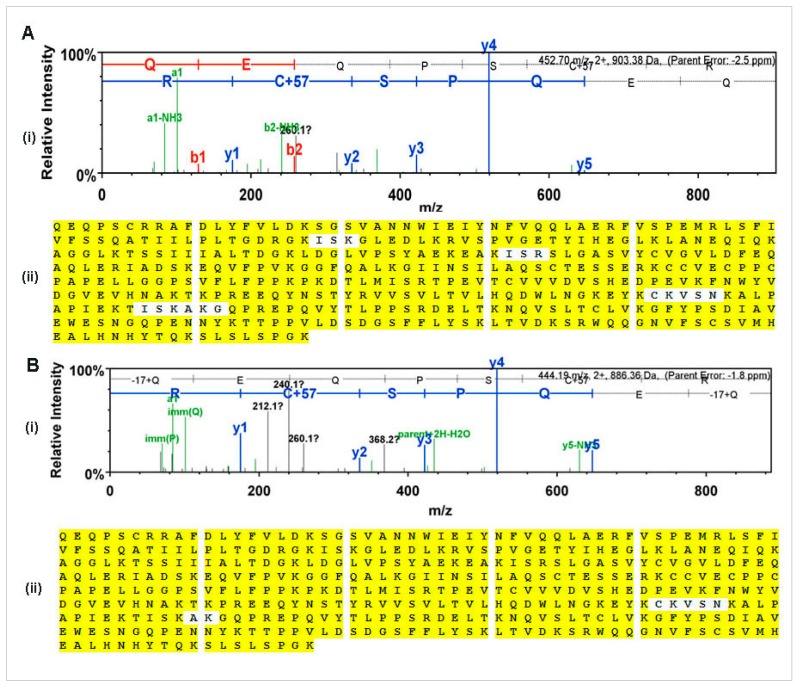
Mass spectrometry analysis: (**A**) rCMG2-Fc-Apo purified from whole leaf extract (**B**) rCMG2-Fc-Apo purified from apoplast wash fluid. (**i**): Unique peptide QEQPSCR was identified from the *N*-terminal region of mature protein purified from whole leaf extract and apoplast wash fluid. The *b* ions shown on the spectrum extend from the *N*-terminus and *y* ions shown on the spectrum extend from the *C*-terminus of the tryptic peptides. The colors indicate the loss of ammonia or water from either *b* or *y* ions (green), doubly charged *b* ions (red) and doubly charged *y* ions (blue). (**ii**): Peptide coverage of rCMG2-Fc-Apo protein. The white color background indicates the uncovered region and yellow color background indicates the peptide coverage.

To confirm the authenticity of rCMG2-Fc-Apo protein purified from whole leaf extract and apoplast wash fluid, proteins were subjected to LC-MS-MS analysis. We observed a peptide coverage of 96% and 98% corresponding to whole leaf extract and apoplast wash fluid, respectively (Figure 5A(ii),B(ii)). Also, 155 unique peptides from whole leaf extract and 183 unique peptides from apoplast wash fluid were observed. To confirm the *N*-terminus of the mature protein, the unique peptide QEQPSCR was observed in both samples (Figure 5A(i),B(i)). Based on the MS analysis we confirmed that the rCMG2-Fc-Apo protein purified from whole leaf extract and apoplast wash fluid was identical to the expressed sequence.

### 2.6. N-Glycan Analysis

**Figure 6:**
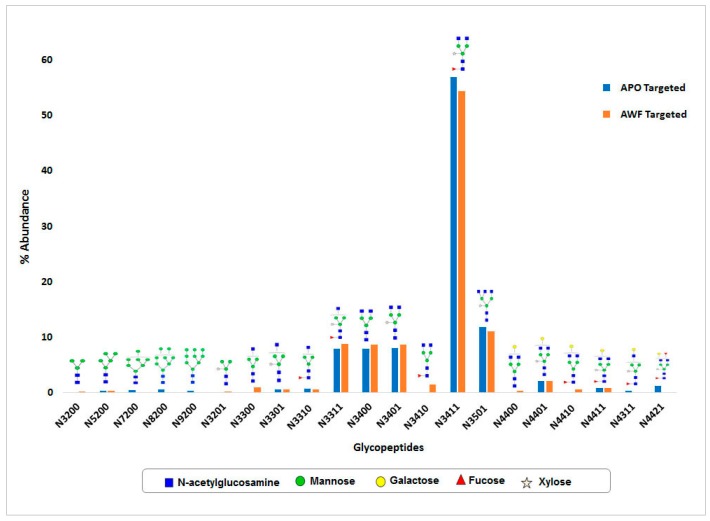
*N*-glycan distribution analysis of rCMG2-Fc-Apo purified from whole leaf extract (APO) and apoplast wash fluid (AWF). Different glycan structures observed at the *N*-linked glycosylation site on the Fc region. The abundance of each glycan structure is represented in a bar chart. The annotation on the *x*-axis represents *N*-glycan-number of Mannose and Galactose residues/number of GlucNAc residues/number of Fucose residues/number of Xylose residues.

The *N*-glycosylation pattern of apoplast targeted rCMG2-Fc-Apo protein from *Nicotiana benthamina* plant biomass was estimated by LC-MS-MS analysis. The analysis revealed a mixture of 15 and 16 *N*-glycan structures for the purified rCMG2-Fc-Apo protein from whole leaf extract and apoplast wash fluid (Figure 6), respectively. In both samples, the major proportion of *N*-glycans consists of complex type structures. The most abundant structure of GlcNAc_2_(Xy_l_)Man_3_(Fuc)GlcNAc_2_ in whole leaf extract (57%) and apoplast wash fluid (54%) was observed. As expected, high mannose *N*-glycan structures were not detected in the apoplast wash fluid preparation. This result is in agreement with our MDH enzymatic assay, which shows there was no intracellular protein leakage in the apoplast wash fluid. Consequently, rCMG2-Fc-Apo protein purified from apoplast wash fluid is secreted by Ramy3D signal peptide.

## 3. Discussion

Currently, there are few US Food and Drug Administration (FDA) approved vaccines and drugs available to treat anthrax infection. BioThrax has been an FDA-approved vaccine since 1970 as a pre-exposure protection against anthrax. This vaccine is produced from a culture filtrate of a non-virulent *Bacillus anthracis* strain. BioThrax is administered by subcutaneous injection of six doses per treatment [25,26]. Raxibacumab is a monoclonal antibody (human γ IgG1) produced by rDNA technology in a murine cell expression system. In December 2012, the FDA approved this drug to treat inhalational anthrax infection [27,28]. Similarly, Obiltoxaximab is another monoclonal antibody, which has been designed to neutralize the free protective antigen. Intravenous administration of this monoclonal antibody for the treatment of inhalational anthrax infection was approved by FDA in March 2016. Intramuscular formulation of this antibody has also been evaluated in healthy human volunteers in a Phase I clinical study [29].

Although FDA approved vaccines are currently available against anthrax disease, anthrax spores are a threat agent of biological warfare and terrorist attacks, and due to the well documented emergence of antibiotic-resistant pathogens, novel drug development remains an unmet need to improve the treatment efficacy and to reduce the manufacturing cost of mass production. In this report, we describe the successful production of rCMG2-Fc-Apo protein in *Nicotiana benthamiana* plants. This Fc fusion protein construct was designed under the control of *CaMV* 35S promoter together with a Ω leader sequence. Transient expression performed by co-infiltration of the gene silencing suppressor p19 was able to achieve a level of >800 mg/kg of this protein-based therapeutic from the infiltrated leaf biomass within six days post-infiltration. These result are in agreement with earlier studies, which showed that co-infiltration of p19 gene silencing suppressor is able to enhance the production of endoplasmic reticulum retained CMG2-Fc protein [30]. Also, co-infiltration of p19 with aglycosylated CMG2-Fc fusion protein also showed a similar effect [31].

Since the first report in 1989 of a CD4-Fc-fusion protein that inhibited entry of human immune deficiency virus into T cells, Fc-fusion technology has been intensely explored for its efficacy to control a variety of human pathologies and other clinical diseases [32]. Fc-based fusion proteins are composed of an immunoglobulin Fc domain that is covalently attached to the protein of interest through a linker peptide. The major advantage of including an Fc-domain is to significantly increase the serum half-life through pH-dependent binding with neonatal Fc receptor, which salvages the endosomal degradation and renal clearance of therapeutic proteins. From a manufacturing perspective, the Fc-domain allows for an easy and cost-effective purification during production in any currently available host system by employing Protein A affinity chromatography. Additionally, the Fc domain can improve the solubility and stability of the therapeutic protein both in vitro and in vivo by independent protein folding [33,34].

Using the Fc fusion technology, we were able to isolate the CMG2 protein from *Nicotiana benthamiana* plant biomass with one step Protein A affinity chromatography at high purity. Downstream bioprocessing of biopharmaceuticals represents some challenge in bioprocess development and its cost is estimated to range from 65% to 90% of total manufacturing costs [35]. Recent developments in protein purification from tobacco plants have improved the cost effectiveness of recombinant protein purification. Several approaches have recently been applied to improve recombinant protein stability by limiting protease activity, such as the application of protease inhibitors to reduce protease activity or recombinant protein targeted in various sub-cellular compartments [36]. In this study, the rCMG2-Fc-Apo protein was targeted to apoplast with the help of the Ramy3D signal peptide, and ethylenediaminetetraacetic acid (EDTA) was used as a protease inhibitor in the extraction buffer. *N*-glycan and MS-MS peptide fingerprinting indicate that the Ramy3D signal peptide was able to effectively secrete rCMG2-Fc-Apo protein to the apoplast. Also, using EDTA as a protease inhibitor we were able to purify rCMG2-Fc-Apo protein with minimal proteolytic cleavage. The MS-MS analysis of purified rCMG2-Fc-Apo protein from whole leaf extract and apoplast fluid revealed the molecule integrity.

Proper *N*-glycosylation is essential for aspects of monoclonal antibody or Fc-fusion protein functionality because the oligosaccharides attached to Fc fragments at the Asn297 position are known to influence binding to cellular Fc receptors strongly and consequently, influence in vivo functionalities [37]. The advantages of using plant-based expression platforms include glycan homogeneity compared with other hosts, ease of large-scale production, and production speed [38,39]. In this study we have targeted rCMG2-Fc-Apo protein for secretion to the apoplast compartment, and *N*-glycosylation of this protein was observed. Around 15 *N*-glycan structural variants were identified from both whole leaf extract and apoplast fluid. In both samples, the major portion of *N*-glycans consists of complex type structures, particularly the GlcNAc_2_(Xy_l_)Man_3_(Fuc)GlcNAc_2_ structure which was most abundant. As expected, high mannose *N*-glycan structures were not detected in the apoplast wash fluid preparation, which indicates the efficiency of protein *N*-glycosylation and rapid secretion. Hamorsky et al. [40] (2015) reported that the overexpression of an aglycosylated CTB (cholera toxin B subunit) by agroinfiltration caused massive tissue necrosis; interestingly, this effect was relieved with the re-introduction of the *N*-glycosylation site. Moreover, high-level protein expression has been observed when the protein is glycosylated and targeting for secretion. Similarly, our rCMG2-Fc-Apo protein showed higher expression levels than for endoplasm reticulum-retained or aglycosylated CMG2-Fc [30,31].

## 4. Materials and Methods

### 4.1. Construction of Binary Vector to Express rCMG2-Fc-Apo Fusion Protein

A gene coding for the human *CMG2* extracellular domain followed by a hinge and two serines and then the coding sequence of the Fc domain from human IgG1 was codon-optimized for *Nicotiana benthamiana* expression. A nucleotide sequence encoding the Ramy3D signal peptide was fused to the *N*-terminal of the *CMG2* coding region to enable secretion to the plant apoplast. Also, nucleotides encoding the Ω leader sequence were included between the start of the coding region and the *CaMV* 35S promoter to improve rCMG2-Fc-Apo protein expression. The binary expression vector (pDP16.0707.07) that was created as a consequence was transformed into *Agrobacterium tumefaciens* EHA105 via electroporation. A binary vector capable of expressing P19 to suppress RNAi-mediated gene silencing in *Nicotiana benthamiana* plants was co-infiltrated with the rCMG2-Fc-Apo binary vector as previously described [30].

### 4.2. Preparation of Nicotiana benthamiana Plants

Wild-type *Nicotiana benthamiana* seedlings were grown from seed in soil-filled 4-inch pots in the greenhouse. Two weeks after germination, seedlings were transplanted into 4-inch pots and the soil was supplemented with Osmocote fertilizer (Scotts Miracle-Gro Company, Marysville, OH, USA). All of the *Nicotiana benthamiana* plants were grown in the greenhouse with a 16-h photoperiod with the optimal temperature of 30 °C (daytime high) and 18 °C (night time low) for four to six weeks.

### 4.3. Bacterial Preparation

Recombinant *Agrobacterium tumefaciens* cells were grown overnight in 10 mL of Luria-Bertani (LB) broth containing appropriate selection antibiotics. For each culture, 2.5 mL was then transferred to 1000 mL flasks containing 250 mL of LB media and grown overnight at 28 °C with 250 rpm shaking. Bacterial cells were harvested by centrifugation at 2600× *g* for 30 min and resuspended in sterile 10 mM MES buffer (pH 5.6) (Fisher Scientific, Santa Clara, CA, USA) containing 10 mM MgCl_2_ and 150 µM acetosyringone (Sigma-Aldrich, St. Louis, MO, USA). The cell density of the resuspended agrobacterial strains was adjusted to achieve an OD_600_ of 0.5 for rCMG2-Fc-Apo and p19 strains. These agrobacterial strains were mixed in a 1:1 volume ratio and were incubated in the dark for up to 3 h before infiltration.

### 4.4. Agroinfiltration and Plant Incubation

Five-week old potted greenhouse *Nicotiana benthamiana* plants were inverted and immersed in 1000 mL of the agrobacterial solution having 0.02% of Silwet-L-77 (Lehle Seeds, Round Rock, TX, USA) and placed in a Nalgene container for vacuum infiltration (−25 in Hg) for 2 min before releasing the vacuum. The infiltrated plants were incubated in a controlled environmental growth chamber at 90% humidity and 21 °C for six days, and then the leaves were cut at the petioles and harvested. The agroinfiltrated leaves were stored at −80 °C for further analysis or were immediately processed to recover apoplast wash fluid.

### 4.5. Extraction and Protein Purification

To determine the production level of rCMG2-Fc-Apo protein at different post-infiltration time points, the biomass was ground in liquid nitrogen at 1:10 ratio (1 g biomass in 10 mL buffer), while for protein purification, a 1:4 (1 g biomass in the 4 mL buffer) ratio was used. Biomass was extracted using phosphate buffered saline (PBS) buffer containing 1 mM EDTA and 2 mM sodium metabisulfite, and incubated for 30 min prior to centrifugation at 2600× *g* for 30 min. The centrifuged samples were filtered through a 0.22 µm filter. Then microfiltered plant extract was purified by Protein-A affinity chromatography (MabSelect SuRe^TM^, GE Healthcare, Marlborough, MA, USA). Two milliliters of Protein-A affinity resin was equilibrated with 10 column volumes of PBS buffer followed by sample load at 1 mL/min flow rate. Then the resin was washed with 15 column volume of PBS buffer. Elution took place by passing 10 column volumes of 100 mM glycine buffer (pH 2.5) to recover the bound rCMG2-Fc-Apo protein. Finally, elution fractions were neutralized with 0.5 M Tris buffer.

### 4.6. Apoplast Wash Fluid Recovery

*Nicotiana benthamiana* plant leaves were harvested post-infiltration and leaves were submerged in harvest buffer consisting of PBS buffer (pH 7.4), 1 mM EDTA, 2 mM sodium metabisulfite, and 0.02% Silwet L-77. The submerged leaves were then placed in a Nalgene container for vacuum application (−25 in Hg) for 2 min before releasing the vacuum. The infiltrated leaves were placed in 50 mL Falcon tubes and centrifuged for 15 min at 4 °C at 900× *g*. The apoplast wash fluid was recovered, filtered through a 0.22 µm filter, and stored at −80 °C.

### 4.7. Malate Dehydrogenase (MDH) Activity Assay

To determine the extent of cellular leakage into apoplast wash fluid, MDH activity assay was performed as described earlier [41]. A standard curve was produced from 0.75 mM β-nicotinamide adenine dinucleotide and reduced dipotassium salt (NADH) (Sigma-Aldrich) diluted in PBS buffer. Apoplast wash fluid was recovered by submerging the leaves in PBS buffer (pH 7.4) at a 1:10 (biomass/buffer) ratio, and applying a vacuum (−25 in Hg) for 2 min before releasing the vacuum. The apoplast wash fluid was recovered from the leaves by centrifugation for 15 min at 4 °C at 900× *g*. MDH activity was measured by adding 100 µL sample in a 96-well plate at room temperature. The reaction was started when 50 µL of 1.5 mM NADH and 50 µL of 2 mM oxaloacetic acid (OAA) (Sigma-Aldrich) were added. The decrease in absorbance at 340 nm in the sample wells was monitored for five minutes with a SpectraMax 340C spectrophotometer (Molecular Devices, Sunnyvale, CA, USA). NADH was used to generate a standard curve and the detection limit for MDH enzymatic activity assay was 0.03 U/mL.

### 4.8. ELISA Analysis

The production level of rCMG2-Fc-Apo protein in *Nicotiana benthamiana* leaves was quantified using ELISA. Microplate wells (Costar 3590, Union City, CA, USA) were coated with Protein-A of *Staphylococcus aureus* (Southern Biotech, Birmingham, AL, USA) diluted to 50 µg/mL in PBS Buffer (pH 7.4) and incubated for 1 h at 37 °C. Blocking was achieved with 5% nonfat dry milk prepared in PBS buffer using a 15-min incubation. After incubation, plates were washed three times with phosphate buffered saline tween-20 (PBST), samples and controls were diluted in PBS buffer, and 50 µL of each sample was applied directly to the coated wells. A standard curve was generated with 2.3, 6.9, 20.6, 61.7, 185.2, 555.6, 1666.7, and 5000 ng/mL using pure CMG2-Fc protein (supplied by Planet Biotechnology, Inc., Hayward, CA, USA) diluted in PBS buffer. Microplates were incubated with 50 µL of diluted samples and standards at 37 °C for 1 h. Then plates were washed three times with PBST buffer and Goat anti-human IgG secondary antibody conjugated with horseradish peroxidase (Southern Biotech) diluted 1:2000 in PBS buffer, which was added. The microplate was incubated for 1 h at 37 °C. Detection was performed with 3,3′,5,5′-tetramethylbenzidine (TMB) substrate (Promega, Madison, WI, USA) and the reaction was stopped with 1 N HCl. Finally, the absorbance was measured at 450 nm with a SpectraMax 340C spectrophotometer (Molecular Devices). Each assay was performed in triplicate, and rCMG2-Fc-Apo protein concentrations were interpolated from the linear portion of the standard curve.

### 4.9. SDS-PAGE and Immunoblot Analysis

Protein samples were diluted with 4× Laemmli buffer (Bio-Rad, Hercules, CA, USA) and heated for 5 min at 95 °C with 5% β-mercaptoethanol (Bio-Rad) for the reducing gel and without β-mercaptoethanol for the non-reducing gel analysis. Electrophoresis was performed for 35 min at 200 V using 4%–20% gradient gel (Bio-Rad). After the electrophoresis completion, gels were washed three times with DDH_2_O and stained in Coomassie Brilliant Blue G-250 (Bio-Rad) followed by destaining in DDH_2_O for overnight. Immunoblot analysis was performed by transferring the gel to a 0.45 µm nitrocellulose membrane (Bio-Rad) at 100 V for 90 min. Blots then washed with PBST buffer and blocked with 5% non-fat dry milk (NFDM) prepared in PBS buffer for overnight at 4 °C. The blot was incubated with 1:2500 dilution of goat anti-human IgG antibody conjugated with alkaline phosphatase (Southern Biotech) for one hour at room temperature. The blot washed three times with PBST buffer and developed using AP conjugate substrate kit (Bio-Rad).

### 4.10. Protein Identification by LC-MS/MS

Ten µg of purified rCMG2-Fc-Apo protein were loaded onto a 4%–20% gradient gel (Bio-Rad). After staining the gel in Coomassie Brilliant Blue G-250 (Bio-Rad) and rinsing in water, the rCMG2-Fc-Apo protein band was excised from the gel for LC-MS/MS based protein identification. Briefly, the protein was digested with sequencing grade trypsin as per the manufacturer’s recommendations (Promega). Peptides were dried using vacuum concentrator and resolubilized in 2% acetonitrile/0.1% trifluoroacetic acid. Peptides were analyzed by LC-MS/MS on a Thermo Scientific Q Exactive Orbitrap Mass Spectrometer in conjunction Proxeon Easy-nLC II HPLC and Proxeon nanospray source. The digested peptides were loaded on a Magic C18 200 Å 3U reverse phase column (75-micron × 150 mm) and eluted using a 90-min gradient with a flow rate of 300 nL/min. An MS survey scan was obtained for the *m*/*z* range 300–1600, spectra of MS/MS were developed using a top 15 method. An isolation mass window (2.0 *m*/*z*) was used for the precursor ion selection, and normalized collision energy (27%) was used for fragmentation. Tandem MS spectra were extracted and charge state deconvoluted by Proteome Discoverer (Thermo Scientific, Asheville, NC, USA).The MS/MS samples were analyzed using X! Tandem (The GPM, thegpm.org; version TORNADO (2013.02.01.1). X! Tandem was set up to search UniProt-*Nicotiana benthamiana*_database (20140416, 1538 entries), the cRAP database of common laboratory contaminants (www.thegpm.org/crap; 114 entries), plus an equal number of reverse protein sequences assuming the trypsin enzyme digestion. Scaffold Proteome Software version 4.0.6.1 (Portland, OR, USA) was used to confirm protein identifications. X! Tandem identifications required at least -Log (Expect Scores) scores of greater than 1.2 with a mass accuracy of 5 ppm. Protein identifications were accepted if they contained at least two identified peptides. Using the parameters above, the Decoy False Discovery Rate (FDR) was calculated to be 4.5% on the protein level and 1.94% on the spectrum level. Proteins that contained similar peptides and could not be differentiated based on MS/MS analysis alone were grouped to satisfy the principles of parsimony.

### 4.11. Site Specific N-Glycan Analysis

Trypsin digestion was carried out using sequencing grade modified trypsin (Promega). First, the samples in 50 mM NH_4_HCO_3_ were denatured and reduced with 2 µL of 550 mM dithiothreitol (DTT) at 65 °C for 50 min. The samples were then alkylated with 4 µL of 450 mM iodoacetamide (IAA) for 30 min, in the dark. One microgram of trypsin in 10 µL of 50 mM NH_4_HCO_3_ was added, and the digestion was allowed to continue in a 37 °C water bath for 18 h. The digestion was subsequently stopped by placing the samples in −20 °C for 1 h. The digested samples were analyzed using an Agilent 1290 infinity UPLC system coupled to an Agilent 6490 triple quadrupole mass spectrometer (QQQ) (Agilent Technologies, Santa Clara, CA, USA). An Agilent Eclipse plus C18 column (RRHD 1.8 µm, 2.1 × 100 mm) connected to an Agilent Eclipse plus C18 pre-column (RRHD 1.8 µm, 2.1 × 5 mm) was used for UPLC separation. A 10-min binary gradient consisting of solvent A of 3% acetonitrile, 0.1% formic acid; solvent B of 90% acetonitrile, 0.1% formic acid in nanopure water (*v*/*v*) at a flow rate of 0.5 mL/min was applied. Analytes were monitored as they were eluting from the LC using dynamic multiple reaction monitoring (MRM). The instrument was operated at a unit resolution in positive ion mode. The MRM results were analyzed using Agilent MassHunter Quantitative Analysis B.05.02 software. The unique glycopeptide mass and the diagnostic glycan oxonium fragments *m*/*z* 204.08 and 366.14 were used to quantify individual glycopeptides. Each glycopeptide concentration (in ion counts) was normalized to the total glycopeptides ion count in the sample.

## 5. Conclusions and Future Prospects

A glycosylated rCMG2-Fc-Apo fusion protein was transiently produced in *Nicotiana benthamiana* at a high level and was efficiently secreted to the apoplast. Purification of rCMG2-Fc-Apo protein from *Nicotiana benthamiana* leaf tissue was achieved by one-step Protein A affinity chromatography, and biophysical characterization of this protein revealed sequence confirmation and molecule integrity. The serum half-life of glycoprotein is mainly based on the presence of terminal sialic acid. *N*-glycosylation analysis of rCMG2-Fc-Apo molecule showed mostly complex type *N*-glycans, which will be a useful starting point for glycan remodeling to produce sialylated glycoforms by in vitro enzymatic approaches.

## References

[1] Sari T., Koruk S.T. (2015). Cutaneous anthrax in an unusual location: Case report. Infez. Med..

[2] Azarkar Z., Zare Bidaki M. (2016). A case report of inhalation anthrax acquired naturally. BMC Res. Notes.

[3] Beyer W., Turnbull P.C.B. (2009). Anthrax in animals. Mol. Asp. Med..

[4] Dixon T.C., Meselson M., Guillemin J., Hanna P.C. (1999). Anthrax. N. Engl. J. Med..

[5] Sweeney D.A., Hicks C.W., Cui X., Li Y., Eichacker P.Q. (2011). Anthrax infection. Am. J. Respir. Crit. Care Med..

[6] Watson A., Keir D. (1994). Information on which to base assessments of risk from environments contaminated with anthrax spores. Epidemiol. Infect..

[7] Goel A.K. (2015). Anthrax: A disease of biowarfare and public health importance. World J. Clin. Cases.

[8] Christopher G.W., Cieslak T.J., Pavlin J.A., Eitzen E.M. (1997). Biological warfare. A historical perspective. JAMA.

[9] Davenport M. (2016). After amerithrax: The state of modern biodefense. Chem. Eng. News.

[10] Swartz M.N. (2001). Recognition and management of anthrax—An update. N. Engl. J. Med..

[11] Carr K.A., Lybarger S.R., Anderson E.C., Janes B.K., Hanna P.C. (2010). The role of *Bacillus anthracis* germinant receptors in germination and virulence. Mol. Microbiol..

[12] Hanna P. (1998). Anthrax pathogenesis and host response. Curr. Top. Microbiol. Immunol..

[13] Pannifer A.D., Wong T.Y., Schwarzenbacher R., Renatus M., Petosa C., Bienkowska J., Lacy D.B., Collier R.J., Park S., Leppla S.H. (2001). Crystal structure of the anthrax lethal factor. Nature.

[14] Petosa C., Collier R.J., Klimpel K.R., Leppla S.H., Liddington R.C. (1997). Crystal structure of the anthrax toxin protective antigen. Nature.

[15] Bradley K.A., Mogridge J., Mourez M., Collier R.J., Young J.A. (2001). Identification of the cellular receptor for anthrax toxin. Nature.

[16] Scobie H.M., Rainey G.J., Bradley K.A., Young J.A. (2003). Human capillary morphogenesis protein 2 functions as an anthrax toxin receptor. Proc. Natl. Acad. Sci. USA.

[17] Santelli E., Bankston L.A., Leppla S.H., Liddington R.C. (2004). Crystal structure of a complex between anthrax toxin and its host cell receptor. Nature.

[18] Pimental R.A., Christensen K.A., Krantz B.A., Collier R.J. (2004). Anthrax toxin complexes: Heptameric protective antigen can bind lethal factor and edema factor simultaneously. Biochem. Biophys. Res. Commun..

[19] Deuquet J., Lausch E., Superti-Furga A., van der Goot F.G. (2012). The dark sides of capillary morphogenesis gene 2. EMBO J..

[20] Scobie H.M., Thomas D., Marlett J.M., Destito G., Wigelsworth D.J., Collier R.J., Young J.A., Manchester M. (2005). A soluble receptor decoy protects rats against anthrax lethal toxin challenge. J. Infect. Dis..

[21] Sharma S., Thomas D., Marlett J., Manchester M., Young J.A. (2009). Efficient neutralization of antibody-resistant forms of anthrax toxin by a soluble receptor decoy inhibitor. Antimicrob. Agents Chemother..

[22] Thomas D., Naughton J., Cote C., Welkos S., Manchester M., Young J.A. (2012). Delayed toxicity associated with soluble anthrax toxin receptor decoy-ig fusion protein treatment. PLoS ONE.

[23] Ashkenazi A., Chamow S.M. (1997). Immunoadhesins as research tools and therapeutic agents. Curr. Opin. Immunol..

[24] Koprowski H. (2005). Vaccines and sera through plant biotechnology. Vaccine.

[25] Hopkins R.J., Howard C., Hunter-Stitt E., Kaptur P.E., Pleune B., Muse D., Sheldon E., Davis M., Strout C., Vert-Wong K. (2014). Phase 3 trial evaluating the immunogenicity and safety of a three-dose biothrax(r) regimen for post-exposure prophylaxis in healthy adults. Vaccine.

[26] Malkevich N.V., Basu S., Rudge T.L., Clement K.H., Chakrabarti A.C., Aimes R.T., Nabors G.S., Skiadopoulos M.H., Ionin B. (2013). Effect of anthrax immune globulin on response to biothrax (anthrax vaccine adsorbed) in New Zealand white rabbits. Antimicrob. Agents Chemother..

[27] Tsai C.W., Morris S. (2015). Approval of raxibacumab for the treatment of inhalation anthrax under the Us Food and Drug Administration “animal rule”. Front. Microbiol..

[28] Migone T.S., Subramanian G.M., Zhong J., Healey L.M., Corey A., Devalaraja M., Lo L., Ullrich S., Zimmerman J., Chen A. (2009). Raxibacumab for the treatment of inhalational anthrax. N. Engl. J. Med..

[29] Greig S.L. (2016). Obiltoxaximab: First global approval. Drugs.

[30] Arzola L., Chen J., Rattanaporn K., Maclean J.M., McDonald K.A. (2011). Transient co-expression of post-transcriptional gene silencing suppressors for increased in planta expression of a recombinant anthrax receptor fusion protein. Int. J. Mol. Sci..

[31] Wycoff K.L., Belle A., Deppe D., Schaefer L., Maclean J.M., Haase S., Trilling A.K., Liu S., Leppla S.H., Geren I.N. (2011). Recombinant anthrax toxin receptor-Fc fusion proteins produced in plants protect rabbits against inhalational anthrax. Antimicrob. Agents Chemother..

[32] Capon D.J., Chamow S.M., Mordenti J., Marsters S.A., Gregory T., Mitsuya H., Byrn R.A., Lucas C., Wurm F.M., Groopman J.E. (1989). Designing CD4 immunoadhesins for aids therapy. Nature.

[33] Czajkowsky D.M., Hu J., Shao Z., Pleass R.J. (2012). Fc-fusion proteins: New developments and future perspectives. EMBO Mol. Med..

[34] Beck A., Reichert J.M. (2011). Therapeutic Fc-fusion proteins and peptides as successful alternatives to antibodies. MAbs.

[35] Wilken L.R., Nikolov Z.L. (2012). Recovery and purification of plant-made recombinant proteins. Biotechnol. Adv..

[36] Pillay P., Schluter U., van Wyk S., Kunert K.J., Vorster B.J. (2014). Proteolysis of recombinant proteins in bioengineered plant cells. Bioengineered.

[37] Jefferis R. (2012). Isotype and glycoform selection for antibody therapeutics. Arch. Biochem. Biophys..

[38] Loos A., Gach J.S., Hackl T., Maresch D., Henkel T., Porodko A., Bui-Minh D., Sommeregger W., Wozniak-Knopp G., Forthal D.N. (2015). Glycan modulation and sulfoengineering of anti-HIV-1 monoclonal antibody PG9 in plants. Proc. Natl. Acad. Sci. USA.

[39] Strasser R., Altmann F., Steinkellner H. (2014). Controlled glycosylation of plant-produced recombinant proteins. Curr. Opin. Biotechnol..

[40] Hamorsky K.T., Kouokam J.C., Jurkiewicz J.M., Nelson B., Moore L.J., Husk A.S., Kajiura H., Fujiyama K., Matoba N. (2015). *N*-glycosylation of cholera toxin B subunit in *Nicotiana benthamiana*: Impacts on host stress response, production yield and vaccine potential. Sci. Rep..

[41] Kingsbury N.J., McDonald K.A. (2014). Quantitative evaluation of E1 endoglucanase recovery from tobacco leaves using the vacuum infiltration-centrifugation method. BioMed Res. Int..

